# Color Image Retrieval Method Using Low Dimensional Salient Visual Feature Descriptors for IoT Applications

**DOI:** 10.1155/2023/6257573

**Published:** 2023-02-23

**Authors:** Naushad Varish, Priyanka Singh, Prannoy Tugiti, Marella Hima Manikanta, Bhavana Yedlapalli, Abhishree Pappusetty, Hiren Kumar Thakkar, Gajendra Sharma

**Affiliations:** ^1^Department of Computer Science and Engineering, GITAM (Deemed to be University), Hyderabad 502329, Telangana, India; ^2^Department of Computer Science and Engineering, SRM University, Guntur 522302, Andhra Pradesh, India; ^3^Department of Computer Science and Engineering, Pandit Deendayal Energy Univrsity, Gandhinagar 382007, Gujarat, India; ^4^School of Engineering, Department of Computer Science and Engineering, Kathmandu University, Dhulikhel 45200, Kavre, Nepal

## Abstract

Digital data are rising fast as Internet technology advances through many sources, such as smart phones, social networking sites, IoT, and other communication channels. Therefore, successfully storing, searching, and retrieving desired images from such large-scale databases are critical. Low-dimensional feature descriptors play an essential role in speeding up the retrieval process in such a large-scale dataset. A feature extraction approach based on the integration of color and texture contents has been proposed in the proposed system for the construction of a low-dimensional feature descriptor. In which color contents are quantified from a preprocessed quantized HSV color image and texture contents are retrieved from a Sobel edge detection-based preprocessed V-plane of HSV color image using a block level DCT (discrete cosine transformation) and gray level co-occurrence matrix. On a benchmark image dataset, the suggested image retrieval scheme is validated. The experimental outcomes were compared to ten cutting-edge image retrieval algorithms, which outperformed in the vast majority of cases.

## 1. Introduction

Due to the advent of diverse devices such as smart phones, tablets, drones, CCTVs, and other image-capturing tools, as well as high-speed Internet, technology and its range of applications are rapidly expanding in the contemporary era. Huge amounts of unstructured image data have been generated in a variety of disciplines, including medical and health insurance, forensics, cars, archaeology, criminal prevention, architecture, and defence [1–4]. As the Covid effect fades across the world, it is anticipated that roughly 1.4 trillion photos will be taken in 2021. In addition, with the apparent rise in relevance of the IoT, edge devices such as smartphones generate a great quantity of photos. Huge amounts of data necessitated an appropriate approach to organise, manage, and retrieve photographs from a vast database, which proved to be a difficult undertaking. Text-based image retrieval (TBIR) techniques were initially used to retrieve images from digital datasets using text-based queries based on their annotations, which could include a range of descriptions or tags. Because human engagement or labour is an important part of the annotation/tagging process, it highlighted numerous errors. Therefore, traditional procedures are ineffective, and their accuracy is questioned. Furthermore, it is a time-consuming, costly, and repetitive operation. As a result, alternative approaches known as content-based image retrieval (CBIR) have been discovered to overcome the shortcomings of traditional methodologies, providing a fresh opportunity to solve the problem of image retrieval [5]. CBIR is the technique of automatically indexing and retrieving images from big databases based on the contents of the images known as features such as color, texture, and shape. A novel content-based picture retrieval strategy is proposed in the presented work. Unlike previous systems, which rely on a single feature for retrieval, our research uses color and texture content for image retrieval; color and texture are the most important visual features for humans [6]. The color and texture contents of preprocessed photographs were retrieved in this research, and the Laplacian filter was used to remove unnecessary information by sharpening the color images. To extract the image's color instances, the HSV color space quantization approach is being used. The texture contents are obtained with discrete cosine transformation (DCT) and gray level co-occurrence matrix (GLCCM), and the image is then processed with the Sobel edge detection method. The spatial and interblock relationships were determined using GLCCM-based DC and AC coefficients to calculate these contents. Finally, by fruitfully merging color and texture contents, a low-dimensional single feature descriptor is generated, which speeds up retrieval. The Euclidean distance is being used to compute the similarity between the database image feature descriptors and the query image. The accuracy of the proposed scheme was tested using two datasets, the Corel 1K and the UC Merced, in terms of precision rates, recall rates, and F-scores rates.

### 1.1. Motivation

The retrieval of the desired images from a digital repository with semantically varied categories is a very tedious task for many researchers, especially in cloud assisted IoT [7]. The image retrieval is a searching technique in several real-world applications, such as medical imaging [8], searching individual video frames [9], object retrieval [10], image classification [11], digital libraries [12], and multimedia event detection [13]. In this regard, several CBIR schemes have been proposed based on transformation tools like discrete cosine transformation (DCT) and/or spatial domain techniques like color quantization, color histogram to extract the effective and significant features for image retrieval applications [14–16]. In most of the existing CBIR applications, either transformation tool or color information is applied for feature extraction. It is evident that the combination of the DCT and color-based approaches provide the significant image feature descriptors for image retrieval. The proposed research work is motivated by a simple nonuniform histogram quantization process and block level DCT based inter-related information using statistical analysis. In order to extract image information, the statistical color moments from quantized image have been computed while an image plane is divided into 8 × 8 fixed-sized blocks, and each block is employed by the DCT transform to get inter-related information. The main advantage of applying DCT is that it has powerful image analysis and discriminative properties. To improve the performance, the color information-based features are fused with DCT-based features for effective and efficient image retrieval.

## 2. Literature Survey

Numerous CBIR approaches have been presented that are based on the extraction of low-level image features/contents in the transform or spatial domain. Aamer et al. [17] developed a scheme/method for extracting DCT features from images that improves retrieval speed and reduces the amount of storage required during image retrieval. In this study, the researchers separated the input image into 8 × 8 nonoverlapping chunks and then applied DCT to each one. The image features can be extracted from the histograms of the quantized AC and DC coefficients of each transformed block, and the Euclidian distance between the query image's features and the database images can be calculated, and the closest images from the database can be retrieved using the minimal level quantified similarity measures. Yun et al. [18] suggested a CBIR approach based on the image's color and texture attributes. Color features are taken from distinct normalized GCLMs of the grayscale image, while texture features are extracted from both color and block color histograms. For superior retrieval results, they combined both features using a simple fusion method. Kavitha et al. [19] present another block-based image retrieval approach, in which an image will be first segmented into equal-sized sub-blocks for feature extraction. After that, the color information for each block is recovered by dividing the HSV color space into nonequal periods and representing the color features with a cumulative histogram. To represent the final feature, the texture feature is obtained using GLCM and combined with the color feature. Priyanka et al. [20] conducted a comparison of CBIR systems employing various feature extraction approaches. The texture feature was computed using wavelet and Gabor filters, while the color feature was retrieved using the color moments of the HSV color space. The similarity distance is calculated using the chi-square and Euclidean distances, and the top photos with similar features are retrieved. They found that employing Euclidean distance to combine color moments with Gabor texture gave them the highest precision rates of any known method. Jiquan ma et al. [21] suggested a CBIR scheme for image feature extraction based on HSV color space and discrete wavelet transform (DWT). They used the wavelet transform to breakdown the signal into a number of fundamental functions, and then used the Daubechies-4 wavelet transform to decompose the image. To create an eight-dimensional texture feature, the mean and standard deviation of the four bands are determined. The texture feature based on wavelet transform provides a better performance and stability, according to the testing results. Wang et al. [22] proposed image retrieval based on DCT and DWT with feature extraction utilising grading algorithms in 2015. The color moments, color histogram, and a novel dynamic color space quantization based on color distribution were modified to generate a color feature in the DCT domain, while the texture feature was computed using the DWT domain. In terms of retrieval accuracy, the experimental findings show that two grading image retrieval methods operate efficiently and effectively. Kaipravan et al. [5] propose another CBIR approach based on color and texture features. The color feature is computed by partitioning an image into three equal horizonal regions and then computing the two color moments from each subimage plane using each color channel separately. Gabor wavelets capture energy at a given frequency and orientation to extract the texture information. Weights are assigned to each feature vector, and the Manhattan distance is used to calculate the similarity measure. They came to the conclusion that a single color or texture feature is insufficient to effectively characterise a picture; therefore, color-texture features are combined for improved retrieval efficiency. Chen et al. [23] constructed a CBIR technique that extracted color-texture features utilising the HSV color space in the year 2020. For feature representation, they first divided the image into 4 × 4 blocks to split the image into 16 sub-blocks. In order to extract significant features, the proposed method further divides a rectangular overlapping block into nine overlapping sub-block regions based on the sixteen sub-blocks. This overlapping method has advantages such as reducing the storage space and reducing the calculation amount of the similarity measure of the image. This method also does not destroy the information connection between the images because of the sub-blocks, thus ensuring better retrieval accuracy. Our presented work is also compared with some state-of-art schemes; those are described one after another in detail. In year 2015, Shrivastava et al. [24] proposed a new image retrieval technique that retrieves similar images from an image dataset in three stages using primitive low-level image features such as color, texture, and shape. In their proposed scheme, a fixed number of images are first retrieved based on their color feature similarity. The color feature was extracted using quantized color histograms in HSV color space, and the number of pixels in each bin of the histogram was used to form a color feature vector. To improve the retrieved images' relevance, their texture and shape features are matched, respectively. The Gabor wavelet transform was used to compute the texture information of the image, while the shape feature vector was constructed by computing the Fourier descriptor based on centroid distance. This method reduced the computation time and increased the overall accuracy due to the retrieval of images in three different stages. Later in year 2016, Dubey et al. [25] proposed a novel method for image description with multichannel decoded local binary patterns and introduced an adder and decoder-based scheme for combining the local binary patterns (LBPs) from multichannel of image. Two multichannel decoded local binary patterns are introduced-multichannel adder local binary pattern (maLBP) and the multichannel decoder local binary pattern (mdLBP). Both maLBP and mdLBP utilize the local information of multiple channels based on the adder and decoder concepts. The feature descriptor has high dimensionality due to combination of the multichannel-based LBPs. Mistry et al. [26] proposed a hybrid feature-based efficient CBIR scheme using various distance measures in year 2018. Spatial domain features including color auto-correlogram, color moments, HSV histogram, and frequency domain features like moments using SWT and Gabor wavelet transform were used. Further, color and edge directivity descriptor features were performed to enhance precision binarized statistical image features. They claim that their results are better than all the existing models. Similarly, in 2018, Irtaza et al. [27] proposed an approach that resolves the classification disagreement amongst different classifiers and the class imbalance problem in CBIR. They have used a genetic algorithm (GA)-based classifier comity learning (GCCL) method to generate stable classifiers by combining ANN with SVMs through asymmetric and symmetric bagging. Once the stable classifiers were generated, the query image was presented to the trained model to understand the underlying semantic content of the query image for association with the precise semantic class. Later, they computed the feature similarity within the obtained class to generate the semantic response of the system. Later on, in year 2019, Ahmed et al. [28] proposed a novel technique to fuse the spatial color information with shaped extracted features and object recognition which increases the strength of the image features for the information fusion purpose in retrieval process. They extracted the color features from RGB images and used the gray level image for the pixel intensity-based local features. They combined the local image features, spatial information in BoW architecture and evaluated the results on popular image collection databases. Vimina et al. [29] proposed another CBIR scheme in year 2020 using texture-color descriptor by integrating the multichannel features. For the texture feature, they used a fixed-sized local intensity-based descriptor, MMLBP, integrating the multichannel local intensity information of the image at the pixel level. The dimensionality of the descriptor is fixed irrespective of the number of channels in the image. The resulting histogram of the patterns is used for representing the image texture. The color feature is extracted by quantizing the RGB color space and is represented with a histogram. The color-texture descriptors are further fused to characterise the images. The MMLBP, along with a quantized color descriptor, is used for characterising the images for retrieval purpose. Garg et al. [30] proposed a CBIR scheme in year 2021 to obtain feature descriptor from multilevel image decomposition. They achieved this by extracting approximation and correct coefficients by applying discrete wavelet transformation to the RGB channels. Therefore, both approximation and correct coefficients are applied to the dominant rotated local binary pattern called texture descriptor, which is computationally effective and rotationally invariant. The local descriptors are extracted from the entire image, for which they used methods such as SIFT, SURF, HoD, and LBP. A rotation invariance function image for a local neighbor patch is obtained by measuring the descriptor relative to the reference. It navigated approaches that contained the complete structural information, extracted directly from the local binary patterns, and the additional information like the information of magnitude, which, in turn, achieves extra discriminating power. Then, the GLCM description is used by obtaining the dominantly rotated local binary pattern image to extract. The proposed scheme is trained and tested on the three classifiers: support vector machine, K-nearest neighbor, and decision tree. Varish et al. [31] demonstrates an image retrieval scheme where a fused low-dimensional feature descriptor is obtained by fusing probability histogram-based HSV color moments and multiresolution-based shape moments. The color moments and shape moments were extracted from the Laplacian filter-based preprocessed image. In addition to the color-shape feature, the texture feature is also included by Sumit et al. [32] for feature representation, where YCbCr color space for the feature extraction process is used and Y, Cb, and Cr color planes are minimally overlapped. A mid-rise quantization scheme preprocesses the images. The texture and shape features are extracted from the Y plane using BDIP and BVLC techniques. Subsequently, they used adaptive tetrolet transform in the output of BDIP and BVLC to extract local textural and geometrical features. At the same time, they selected the Cb and Cr components and applied adaptive tetrolet transform to analyze the regional local color variations of the image. Finally, they combined the nonoverlapping extracted shape, texture, and color features to form the final feature vector for the retrieval process. In order to, reduce image retrieval's search space and computational complexity, Joseph et al. [33] developed a CBIR scheme that investigates various search space reduction techniques and classifies the image collection into a subset of related images. They proposed an image clustering using hybrid K-means moth flame optimization algorithm (KMFO) which enhances the performance of the K-means algorithm by assigning the optimum number of clusters and cluster centroids based on the number of flames and flame values. HSV color histogram, color correlogram, wavelet transform, GLCM, color moments, dominant color, and region-based descriptors are used as feature descriptors. Motivated by the above-discussed works, authors have proposed a novel feature extraction technique using color and texture features based on the spatial domain and transform domain, respectively, where features have been computed from an HSV color image.

### 2.1. Major Contribution

The main contribution of the proposed image retrieval scheme includes the following:

- A low-dimensional feature descriptor using the fusion of spatial domain-based color information and transform domain-based texture information is constructed for image retrieval applications. It reduces the computational overhead for retrieving images from large-scale datasets in a speedy manner.
- To extract the color information, a nonuniform quantized color histogram is used and subsequently the color moments from the preprocessed image have been computed for formation of color feature descriptor.
- The inter-related information between blocks has been extracted using block level DCT tool, where associativity has been determined using the AC and DC coefficients of image blocks.
- The spatial relationship between the preprocessed AC and DC coefficients in the DCT domain has been established using GLCCM, and subsequently, the statistical parameters have been calculated from corresponding GLCCMs for the construction of texture feature descriptors.
- The integration of color and texture information is done, and the proposed image retrieval scheme is validated on two Corel 1K and the UC Merced benchmark databases, where the diversified results have been achieved.

### 2.2. Organization of Paper

The remainders of the paper are laid out as follows: The suggested CBIR image retrieval approach, as well as its feature extraction and retrieval procedure, is discussed in detail in Section 3. The experimental results and comments are detailed in Section 4, and a comparative study with existing retrieval methods is presented to quantify retrieval efficiency. Finally, Section 5 brings the proposed work to a close.

## 3. Proposed Image Retrieval Methodology

The proposed scheme comprises of preprocessing, color, and texture information, and similarity distance-based image retrieval system. Details of each will be described and discussed in the following subsections.

### 3.1. Preprocessing

The Laplacian filter [31] is considered in the proposed scheme for sharpening color images. Because it is based on second-order derivatives, this filter produces a considerably enhanced version of the image, whereas other kernels such as Prewitt, Sobel, and others are based on first-order derivatives. Fine thin lines and isolated points are also produced. The 3 × 3 mask with centre (−8) has been used in the presented work for filtering (L1) images, as shown in equation (1), and it has been implemented in all spots of the image by a convolving operation. This mask is not just for grayscale images; it may also be used on color images.

(1) $$\begin{array}{c} L1=\left[\begin{array}{ccc} 1 & 1 & 1\\ 1 & -8 & 1\\ 1 & 1 & 1 \end{array}\right]\text{.} \end{array}$$

The HSV color image is decomposed into its three color components: H, S, and V. Color visual characteristics are retrieved from the H and S components of the HSV color image, while texture visual features are computed from the V component. In [34], the entire process of preparing an RGB image is detailed with multiple kernels.

### 3.2. Color Feature Representation

An HSV color image is more intuitive and closer to people's subjective color consciousness than visuals in other color spaces [35]. In this paper, an RGB color image is first converted into the HSV color model, and then some preprocessing processes are performed. This step is important since the RGB color space specifies the image in primary colors, which is less effective than the HSV color space when it comes to describing objects. Similar to how the HSV color space defines an image, the human eye interprets images based on comparisons such as color, vibrancy, and brightness. The values of H, S, and V must be transformed to a specified range based on the human perception system for easier computation. The hue component has angles ranging from 0 to 360 degrees, while the value and saturation components have values ranging from 0 to 1 percent. The image in HSV color space needs to be quantized according to the human eye's perception characteristics as referenced in [36]. The obtained *H*′*S*′*V*′ color image contains only 81 colors which represent original images with lesser numbers of colors as compared to the original HSV color image. The statistical moments-based color information of an image provide the important properties of intensity level distribution like smoothness, uniformity, flatness, contrast, and brightness [37], which improves the retrieval efficiency of system. The proposed color feature representation method computes mean, standard deviation, skewness, and kurtosis from *H*′*S*′*V*′ color image for formation of the color feature descriptor [38]. Let *μ*_*CC*_ be the mean, *σ*_*CC*_ be the standard deviation, *γ*_*CC*_ be the skewness, and *κ*_*CC*_ be the kurtosis of each *CC* color component, where *CC* ∈ {*H*′, *S*′, *V*′}. The statistical moments from the quantized color image are computed as

(2) $$\begin{array}{c} \mu_{CC}=\overset{T}{\underset{i=1}{\sum }}X_{i}P\left(X_{i}\right)\text{,}\\ \sigma_{CC}=\sqrt{\overset{T}{\underset{i=1}{\sum }}{\left(X_{i}-\mu_{CC}\right)}^{2}P\left(X_{i}\right)}\text{,}\\ \gamma_{CC}=\frac{1}{\sigma^{3}}\overset{T}{\underset{i=1}{\sum }}{\left(X_{i}-\mu_{CC}\right)}^{3}P\left(X_{i}\right)\text{,}\\ \kappa_{CC}=\frac{1}{\sigma^{4}}\overset{T}{\underset{i=1}{\sum }}{\left(X_{i}-\mu_{CC}\right)}^{4}P\left(X_{i}\right)\text{,} \end{array}$$

where *X*_*i*_ is the *i*^*th*^ pixel value in the *CC* color component, *𝒫*(*X*_*i*_) is the corresponding probability and *T* is the total number of pixels in the corresponding component. The *μ* is the average of intensity values, which describes the brightness of an image while the *σ* measures the distribution of intensity values about the mean and defines the contrast of an image. The *γ* the measure of symmetry or more precisely about its mean value and it also determines the lack of symmetry in a set of data points. The kurtosis calculates peak of the distribution of intensity values about the *μ* and also measures the outliers present in the distribution. An integration of statistical moments from all three color components has constructed the color feature descriptor, which represents the color information of the image effectively. Hence, color feature descriptor is defined as

(3) $$\begin{array}{c} {FV}_{Color}=\left\{\mu_{CC},\sigma_{CC},\gamma_{CC},\kappa_{CC}\right\}\text{,} \end{array}$$

where *CC* ∈ {*H*′, *S*′, *V*′}, since the four moments have been computed from each color component, therefore, the dimension of the color feature descriptor will be 12. The algorithmic 1 steps for color feature representation are as follows:

### 3.3. Texture Feature Representation

**Figure 1:**
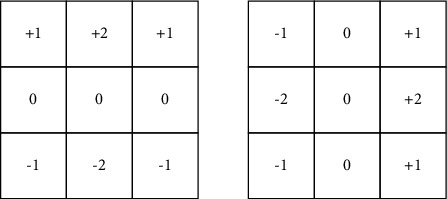
Sobel convolution kernels.

Sobel edge detection ([39]), discrete cosine transformation ([40]), and the gray level co-occurrence matrix (GLCCM) ([41]) are used to extract texture information. It is a derivative-based approximation operator that performs a 2D gradient measurement on an image and accentuates high spatial regions around the edges. As seen in the image in Figure 1, the operator is made up of a pair of 3 × 3 convolution kernels. One kernel is simply the other one 90^0^ rotated. The fundamental goal of edge detection is to reduce the quantity of data in an image while keeping structural qualities that can be used for future image processing. There are several edge detector techniques, and this study concentrates on the Sobel edge detection methodology.

In the proposed method, the Sobel operator is employed on the Laplacian-based preprocessed V-component. The convolved operation is performed image using given template, and the gradient values in each horizontal and vertical directions are computed. These kernels are designed to respond efficiently to the edges running vertically and horizontally relative to the pixel grid. One kernel is considered for each of the two perpendicular orientations noted. These kernels can be used individually on the input image to yield distinct measurements of the gradient component in each orientation as *f*_*a*_(*a*, *b*)=*f*_*a*_ and *f*_*b*_(*a*, *b*)=*f*_*b*_. One can acquire the absolute magnitude of the gradient at each position as well as the gradient's orientation by combining these two. The magnitude of the gradient is calculated as follows:

(4) $$\begin{array}{c} \left|f\right|=\sqrt{f_{a}^{2}+f_{b}^{2}}\text{,} \end{array}$$

where

(5) $$\begin{array}{c} f_{a}=f\left(a-1,b+1\right)+2f\left(a,b+1\right)+f\left(a+1,b+1\right)\\ -\left[f\left(a-1,b-1\right)+2f\left(a,b-1\right)+f\left(x+1,y-1\right)\right]\text{,}\\ f_{b}=f\left(a+1,b-1\right)+2f\left(a+1,b\right)+f\left(a+1,b+1\right)\\ -\left[f\left(a-1,b-1\right)+2f\left(a-1,b\right)+f\left(a-1,b+1\right)\right]\text{.} \end{array}$$

The angle of orientation of the edge (relative to the pixel grid) giving rise to the spatial gradient is given by

(6) $$\begin{array}{c} \theta =\arctan\left(\frac{f_{b}}{f_{a}}\right)\text{,} \end{array}$$

When the orientation is 0, the largest contrast from black to white runs from left to right on the image, and all other angles are measured clockwise from the horizontal direction. The approximate magnitude is determined as follows for easy and quick computation:

(7) $$\begin{array}{c} f=\left|f_{a}\right|+\left|f_{b}\right|\text{.} \end{array}$$

The discrete cosine transform converts an image from the spatial to the frequency domain, and is commonly used in data reduction, feature extraction, and watermarking. The block of size *Y* × *Y* is represented by *f* (p, q). An image's 2-D DCT transform can be defined as shown in the following equation:

(8) $$\begin{array}{c} F\left(s,t\right)=\frac{2}{N}C\left(s\right)C\left(s\right)\overset{Y}{\underset{p=1}{\sum }}\overset{Y}{\underset{q=1}{\sum }}f\left(p,q\right)\alpha \beta \text{,} \end{array}$$

where

(9) $$\begin{array}{c} C\left(s\right)=C\left(t\right)=\left\{\begin{array}{cc} \frac{1}{\sqrt{2}} & if\;s=t=0\text{,}\\ 1 & if\;s,t>0\text{,} \end{array}\right. \end{array}$$

and *α*=cos [((2*m*+1)*uπ*)/(2*N*)], *β*=cos [((2*n*+1)*vπ*)/(2*N*)].

**Figure 2:**
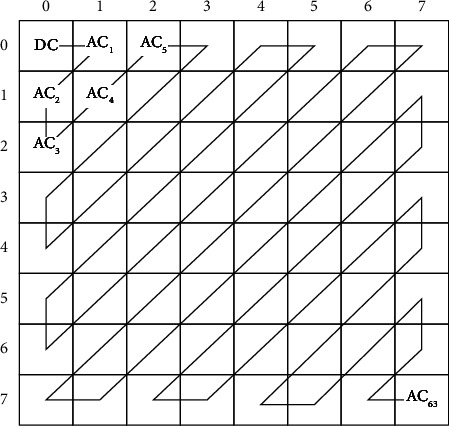
Zigzag scanning order for 8 × 8 image block [40].

The function *𝔉*(*s*, *t*) represents DCT transformed image corresponding to the given image block *f*(*p*, *q*) with respect to the (*p*, *q*) coordinates. The transformed image has DCT coefficients which represents the image information. The most important image information is concentrated in the upper left corner of the transformed image known as the low-frequency band information while the lowest right corner has the insignificant information known as high-frequency band information and it reflects the contour and other unnecessary information of the image. The some coefficients in low-frequency band have been selected by discarding the high-frequency band information completely. The value *𝔉*(0,0) represents DC coefficient or average/energy of image and the remaining DCT coefficients are known as AC coefficients. In order to find the appropriate information, the transformed block is quantized using standard quantization table [42]. Thereafter, the selection of DCT coefficients in zigzag scanning order gives the most appropriate image information, and this order is shown in Figure 2.

Since, the image blocks contains the overlapping or relative information with each other, in order to determine the associativity between the blocks based on the DC and AC coefficients, DC and AC matrices have been established for feature extraction. The process for selection of DCT coefficients and formation of DC and AC matrices are described in the following equation. For two adjacent image blocks,

(10) $$\begin{array}{c} {DC}_{df\;i}=\left|{DC}_{b\left(i+1\right)}-{DC}_{b\left(i\right)}\right|\text{,} \end{array}$$

where *b* represents block, *DC*_*b*(*i*)_ is energy of *i*^*th*^ block and *DC*_*df* *i*_ is absolute difference value between the next block (*i*+1)^*th*^ and current block *i*^*th*^. This value has been computed throughout the image. For example, if size of an image size *M* × *N* and block size is 8 × 8, then total number of blocks will be *bn*=*M* × *N*/8 × 8. So, all the difference values are collected as follows:

(11) $$\begin{array}{c} {DC}_{val}=\left\{{DC}_{df1},{DC}_{df2},\ldots ,{DC}_{df\left(bn-1\right)}\right\}\text{.} \end{array}$$

Similarly, eight values have been selected from each block and special kind of coding is performed on selected AC coefficients in zigzag order between two adjacent blocks. The computation process is given as

(12) $$\begin{array}{c} {AC}^{j}=\left\{\begin{array}{cc} 1 & :\left({AC}_{b\left(i+1\right)}-{AC}_{b\left(i\right)}\right)>0,\\ 0 & :Otherwise\text{,} \end{array}\right. \end{array}$$

where *j* represents number of selected AC coefficients from each block. For one block *AC*^*j*^, *j*=1,2,…, 8, values are given as for example

(*AC*^1^, *AC*^2^, *AC*^3^, *AC*^4^, *AC*^5^, *AC*^6^, *AC*^7^, *AC*^8^)=(1,1,1,1,0,1,1,0)=246 which is the decimal representation and this decimal value is represented by array *AC*_*df* *i*_. Thus, the collected values of an image is given as

(13) $$\begin{array}{c} {AC}_{val}=\left\{{AC}_{df1},{AC}_{df2},\ldots ,{AC}_{df\left(bn-1\right)}\right\}\text{.} \end{array}$$

Finally, the DC and AC matrices have constructed by using (11) and (13), respectively. These matrices will be used for formation of the texture feature descriptor.

The gray level co-occurrence matrix (GLCCM) is one of the important methods to examining the texture properties of the image. To obtain the texture feature descriptor, GLCCM is applied on both DC and AC matrices separately and create GLCM eigenvectors from certain parameters. The GLCCM for a given pair of pixels in a specific direction (*θ*) and particular pixel distance *d* is defined as the frequency of elements *i* and *j* of the matrix, respectively. The value of the GLCCM is denoted by the *P*(*s*, *t*|*d*, *θ*) which is computed symmetrically throughout the matrix and the size of GLCCM is depend on gray levels number. In general, the GLCCM method computes four matrices for four directions i.e., 0^0^, 45^0^, 90^0^, 135^0^ and for a constant pixel distance *d*. For texture feature representation, first GLCCM is normalized to avoid variation among the elements of the matrix. The normalized element *P*(*i*, *j*) of GLCCM is defined as

(14) $$\begin{array}{c} P\left(s,t\right)=\frac{P\left(s,t\left|d,\theta \right.\right)}{\sum_{s=1}^{G}\sum_{t=1}^{G}P\left(s,t\left|d,\theta \right.\right)}\text{,} \end{array}$$

where *G* is the total number of gray levels. In order to extract the texture features, a number of statistical parameters are obtained from normalized GLCM, however, the computational requirements for considering all these are too high and unrealistic. Therefore, only four parameters are considered for construction of texture feature descriptor in this work. The four parameters are defined as

(15) $$\begin{array}{c} Contrast\;F_{con}=\overset{G}{\underset{i=1}{\sum }}\overset{G}{\underset{i=1}{\sum }}{\left(i-j\right)}^{2}P\left(i,j\right)\text{,}\\ Energy\;F_{asm}=\overset{G}{\underset{i=1}{\sum }}\overset{G}{\underset{i=1}{\sum }}P^{2}\left(i,j\right)\text{,}\\ Entropy\;Fent=\overset{G}{\underset{i=1}{\sum }}\overset{G}{\underset{i=1}{\sum }}P\left(i,j\right)log_{2}P\left(i,j\right)\text{,}\\ Correlation\;Fcor=\frac{\sum_{i=1}^{G}\sum_{i=1}^{G}ijP\left(i,j\right)-u_{x}u_{y}}{q_{x}q_{y}}\text{.} \end{array}$$

In the proposed method, above the texture parameters from the gray level co-occurrence matrices *GLCCM*_*DC*_ and *GLCCM*_*AC*_ of the DC and AC-based components, respectively, where 4-4 matrices have been constructed using four directions i.e., 0^0^, 45^0^, 90^0^, 135^0^ and corresponding the energy, contrast, entropy and correlation have been constructed. The collective values of the abovementioned four parameters represent the texture feature descriptor of any image.

(16) $$\begin{array}{c} FV=\left\{F_{con},F_{asm},F_{ent},F_{cor}\right\}\text{,} \end{array}$$

Let *FV*_*DC*_ and *FV*_*AC*_ be represent the texture properties of DC and AC-based matrices and calculated by using (16), then the final texture feature descriptor is obtained as

(17) $$\begin{array}{c} {FV}_{Texture}=\left\{{FV}_{DC},{FV}_{AC}\right\}\text{.} \end{array}$$

The speed and retrieval of the system is improved vastly due to the formation of low dimensional texture feature descriptor. The whole process of computing the texture feature descriptor is presented by the following algorithmic 2 steps.

**Figure 3:**
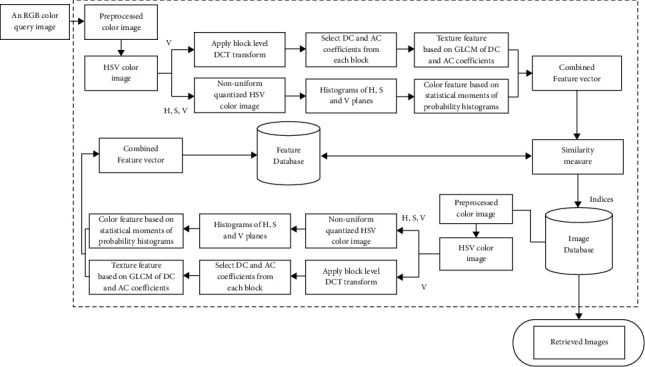
The block diagram of the proposed CBIR scheme.

The block diagram of proposed method is shown in Figure 3, where color and texture features are integrated to present final feature descriptor. The final feature descriptor obtained by integrating the (3) and (17) is given as

(18) $$\begin{array}{c} {FV}_{Final}=\left\{{FV}_{Color},{FV}_{Texture}\right\}\text{.} \end{array}$$

Since this feature descriptor is not normalized because the components are already in a proper range and it is not degrading the retrieval accuracy. The final feature descriptors of all images available in the digital repository and that of the query image are constructed. The collection of final feature descriptors is stored in the feature database, and similarity distances are calculated between the given query and digital repository based on the feature descriptors. Corresponding to the first few minimum distances, the top-desired images have returned to the user in relation to the query.

### 3.4. Similarity Measure

To offer an appropriate response to an image query, a vast number of image databases require both rapid comparison and feature extraction. It can take a long time to look through every image in a huge image library. The appropriate distance between query feature descriptor and database image feature descriptors has been generated to measure the similarity between the query image and the database images. Because of its efficiency and effectiveness, the Euclidean distance is one of the most widely used approaches for retrieval. Therefore, in the proposed system, the Euclidean distance (ED) is used to calculate the similarity measure. It calculates the square root of the total of the squared absolute differences to determine the distance between two image feature descriptors. It is defined as follows:

(19) $$\begin{array}{c} ED=\sqrt{\overset{d}{\underset{i=1}{\sum }}{\left(fd_{q}^{i}-fd_{t}^{i}\right)}^{2},} \end{array}$$

where *d* is the dimension of the feature descriptor, *fd*_*q*_ and *fd*_*t*_ are feature descriptors of query image and target image of the digital repository.

## 4. Experimental Results and Analysis

### 4.1. Database Overview

**Figure 4:**
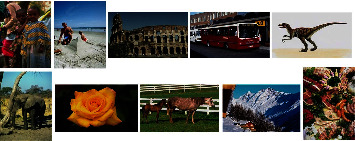
Sample images of Corel-1000 dataset.

**Figure 5:**
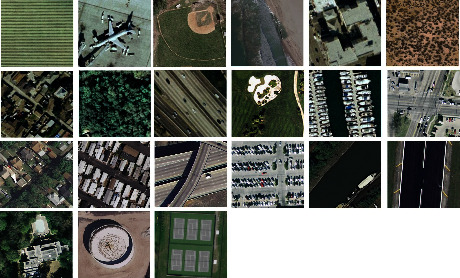
Sample images of UCML radar image dataset.

Two benchmarked datasets were used in the retrieval process to evaluate the efficacy of the proposed CBIR approach. The first is the [43] land-use dataset from UC Merced. It is a typical dataset for image categorization using radar remote sensing that includes 21 different scene types. For the purposes of evaluation, this paper used 900 RGB color photographs divided into nine groups. Agriculture, aviation, roads, grounds, beaches, residential, woodland, chaparral, and harbor are the categories. All of the photos in the collection are 256 × 256 in size, with a resolution of 1 foot. To demonstrate the responsiveness of the retrieval method to changes in image rotation, translation, and size, the image is accessed in JPEG file format. The second is the Corel database [44], which has 1000 RGB color images divided into 10 categories, each with 100 photos of a similar type. People, beaches, buildings, buses, dinosaurs, elephants, roses, horses, mountains, and cuisine are among the image categories. They are in JPEG format and are 256 × 384 and 384 × 256 pixels in size. For both datasets, all of the images were used as query images in the tests. For visualisation, example images of radar and Corel image datasets are shown in Figures 4 and 5, with one image from each category.

In general, any CBIR approach is divided into two parts. In phase I, all of the photos in the dataset are acquired one by one for the extraction of color and texture information. To establish a feature database, the extracted combined features are stored in a database as feature descriptors. In phase II, however, to get comparable types of images from the digital repository using the same way, the user must provide the query as an input image. By computing the similarities using ED, the combined texture and color feature vector is created and compared with the feature descriptors of the database photos. The user gets shown the photographs that are the most comparable to the query image.

### 4.2. Evaluation Measurements

This section has described the performance assessment methods that not only evaluate the effectiveness of image retrieval but also ensure the stability of findings in order to constructively illustrate the success of the suggested image retrieval system. In the purposed CBIR approach, three significant measurements were employed to evaluate retrieval performance: precision, recall, and f-score. These parameters are described as follows:

(20) $$\begin{array}{c} \text{Precision}\left(P\right)=\frac{A}{B}\text{,}\\ \text{Recall}\left(R\right)=\frac{A}{C}\text{,}\\ f-\text{score}\left(F\right)=\frac{2P\times R}{\left(P+R\right)}\text{,} \end{array}$$

where A denotes relevant retrieved images, B denotes the total number of retrieved images from the dataset, and C is the total number of relevant images publicly available per category. Consider the following scenario for clearance: a CBIR technique for query image retrieves a total of 10 images, of which 7 are relevant, from a total of 30 similar images in one database category. The precision will be 7/10 = 70%, while the recall rate will be 7/30 = 21%. Therefore, in this observation it can be observed that the recall rate alone is insufficient to establish the success of a CBIR; precision must also be calculated. F-score in the CBIR method designed for it. These parameters are described as follows: precision and recall are two metrics computed to illustrate the effectiveness of image retrieval, and they assess the accuracy of image retrieval with relevance to the database images and query. These two measurements, however, do not represent the total accuracy of effective image retrieval. So they can be combined to produce a single value that measures picture retrieval accuracy, which is known as the F-Score or F-measure. When retrieving images from the Corel-1000 and UCML-2100 datasets based on a query image, the values of *B* and *C* are set to 10 or 20 and 100, respectively, in the retrieval. Since each image in the category is used as a query image, the accuracy must be presented in terms of averages/means. The average precision, recall, and F-score can be calculated as follows:

(21) $$\begin{array}{c} @P_{avg}\left(M\right)=\frac{1}{nc}\overset{nc}{\underset{k=1}{\sum }}P_{k}\text{,}\\ @R_{avg}\left(M\right)=\frac{1}{nc}\overset{nc}{\underset{k=1}{\sum }}R_{k}\text{,}\\ @F_{avg}\left(M\right)=\frac{1}{nc}\overset{nc}{\underset{k=1}{\sum }}F_{k}\text{,} \end{array}$$

where @*P*_*avg*_(*M*), @*R*_*avg*_(*M*) and @*F*_*avg*_(*M*) are the averages for precision, recall, and F-score, respectively, for *M* image category and *nc* is the total number of images in each category. In this paper, the Corel and radar image datasets are used so the value of *nc*=100 because each image class has 100 images.

### 4.3. Retrieval Results and Discussion

The proposed method is performed on preprocessed and without pre-processed image in the retrieval process using a simple fusion of color and texture descriptors. For color descriptor, an HSV color model is quantized into nonuniform bins to get a new model color *H*′*S*′*V*′ model which consists of 81-colors. Since the four statistical color moments have been computed from each quantized color component, therefore, the dimension of the color descriptor will be 4 × 3=12-D. For texture descriptor, authors have computed DC and AC matrices (i.e., already discussed in texture feature representation section in detail) of *V* component and corresponding to each matrix, the GLCM is computed for four directions i.e., 0^0^, 45^0^, 90^0^, 135^0^ and constant pixel distance (*d*=1). The four values for DC matrix and four values for AC matrix have been computed, so the dimension of the texture feature descriptor is 8-D. The dimension of the fused feature descriptor will be 12 + 8 = 20-D. The fused feature descriptors of all database images have been constructed for retrieving process.  Table 1 shows the top 10 retrieved images from the dataset based on preprocessed and without preprocessed images for UC Merced land-use dataset while same is shown in Table 2 for the top 20 retrieved images. In both the cases, the preprocessed method gives the better results as compare to the without preprocessed image method. In case of top 10 images, it is evident that the preprocessed image technique provides the huge improvement in some category images. For example, the best category image i.e., chaparral has increased precision from 90.52% to 99.50%, which is almost 9% hike from without preprocessed to preprocessed image. Similarly, the worst category image i.e., roads have increased precision from 78.20% to 90.40% which is almost 12% hike from nonprocessed to preprocessed technique. In overall, the mean average for precision, recall, and F-score are satisfactory of the proposed CBIR method for top 10 and 20 retrieved images from the digital repository. It is also noticed from the tables that the accuracy is highly increased from top 10 to top 20 retrieved images.

The F-score results indicate that when using statistical texture features with color different quantization gives the highest retrieval performance for the class chaparral.

**Figure 6:**
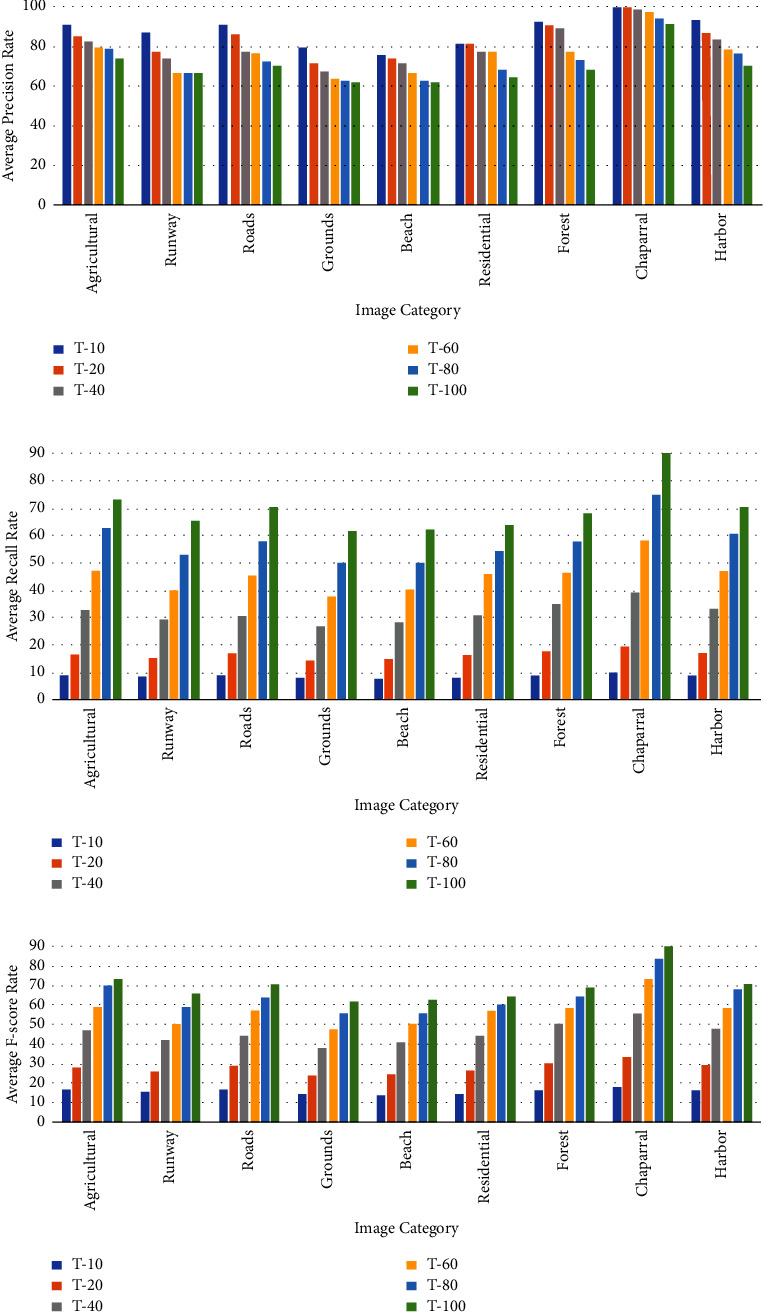
Accuracy in terms of (a) average precision rate (b) average recall rate and (c) average F-score for radar remote sensing image dataset.

**Figure 7:**
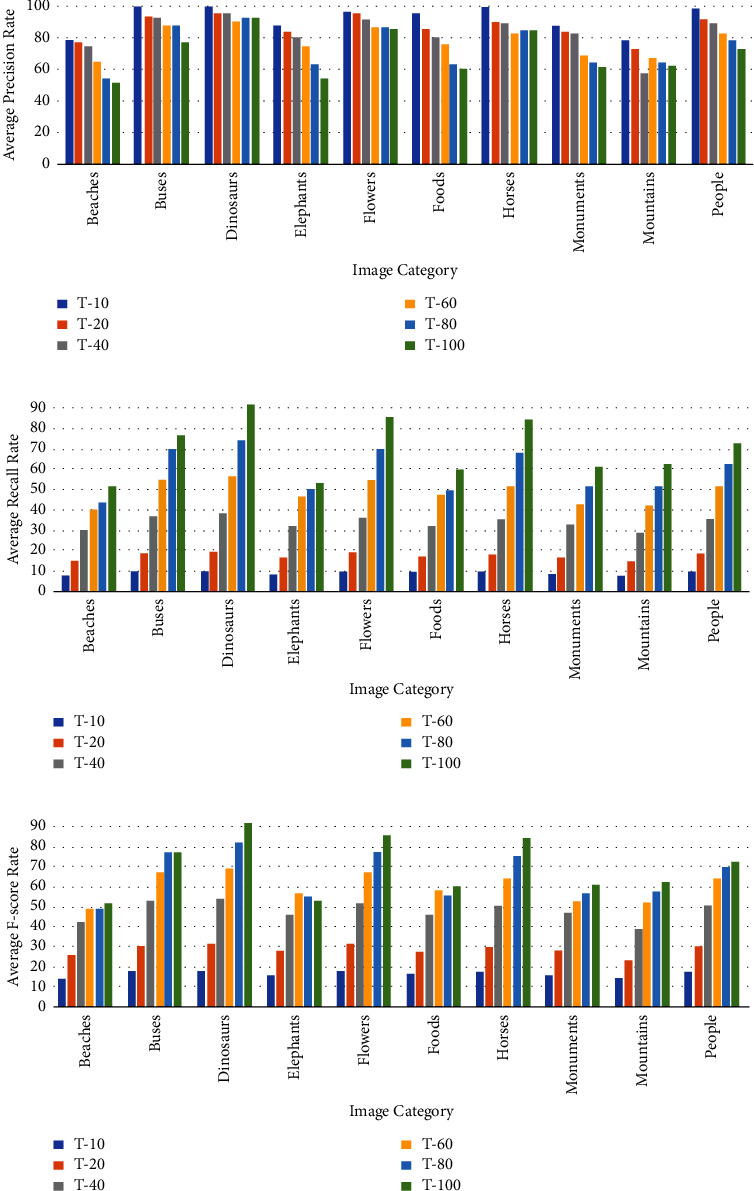
Accuracy in terms of (a) average precision rate (b) average recall rate and (c) average F-score for Corel-1K image dataset.

**Figure 8:**
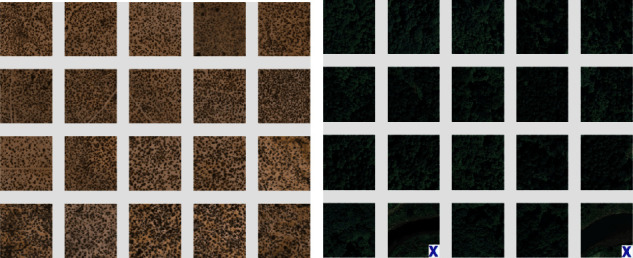
Top 20 retrieved images from radar remote sensing image Corel-1K image dataset. (a) Chaparrals, *P* = 100.00%, *R* = 20.00%, *F* = 33.34%. (b) Forests, *P* = 80.00%, *R* = 18.00%, *F* = 29.38%.

**Figure 9:**
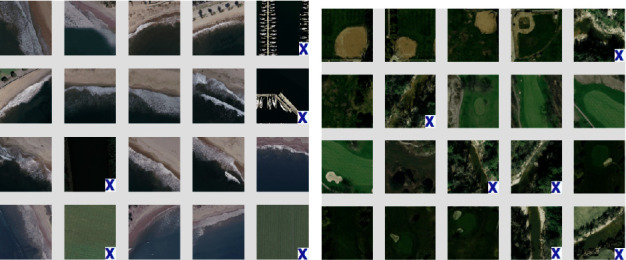
Top 20 retrieved images from radar remote sensing image dataset. (a) Beaches, *P* = 75.00%, *R* = 15.00%, *F* = 25.00%. (b) Grounds, *P* = 70.00%, *R* = 14.00%, *F* = 23.33%.

**Figure 10:**
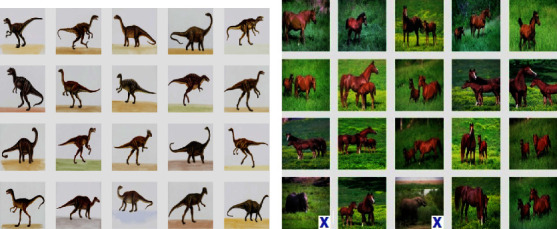
Top 20 retrieved images from Corel-1K image dataset. (a) Dinosaurs, *P* = 100.00%, *R* = 20.00%, *F* = 33.34%. (b) Horses, *P* = 80.00%, *R* = 18.00%, *F* = 29.38%.

**Figure 11:**
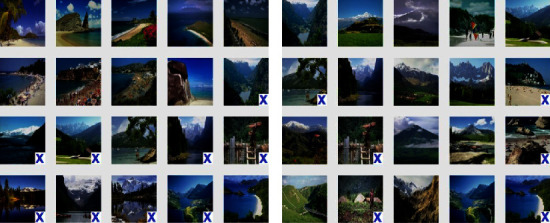
Top 20 retrieved images from Corel-1K image dataset. (a) Beaches, *P* = 55.00%, *R* = 11.00%, *F* = 18.33%. (b) Mountains, *P* = 70.00%, *R* = 14.00%, *F* = 23.33%.

Similarly, the results for the Corel-1K dataset have been included for the top 10 retrieved images using both the preprocessed techniques.  Table 3 shows the retrieval results for the top 10 images using the preprocessed technique for Corel-1K image dataset. In this table, the proposed CBIR method have produced the highest results for dinosaur images while the lowest retrieval results were attained by the mountain category images for the 10 retrieved images. For top 20 images, Table 4 shows the retrieved results, where the most of the category images have good retrieval results but beach and mountains images have the lowest results because the images are complex, structures and their contents are mixed with each other. The overall means for recall, precision, and F-score for top 10 retrieved images without preprocessing are 8.18%, 81.53%, and 14.87%, while it becomes 9.21%, 92.11%, and 16.75% using with pre-processed image. Hence, it is a huge improvement from without preprocessing to with technique. It is also observed from the table that the little bit accuracy has been decreased from top 10 to top 20 images. The retrieval results for top *T* − *i*, (*i* = 10, 20, 40, 60, 80, and 100) images are shown in Figure 6 for radar remote image dataset in terms of average precision, recall, and F-score rate while it is shown in Figure 7 for Corel-1K image dataset, where *T* − *i* represents number of images retrieved from the dataset.For visualisation purposes, we have selected two best and two worst category images from both the radar remote sensing image and Corel-1K image datasets.  Figure 8 shows the best retrieval results for retrieving top 20 images from radar remote sensing image dataset for chaparrals and forest image category, where these results are only given query images. For the chaparral query image, values of the precision (*P*), recall(*R*), and F-score (*F*) are 100.00%, 20.00%, and 33.34%, respectively, while the technique gives *P* = 80.00%, *R* = 18.00%, and *F* = 29.38% for forest image category. For worst-case results, Figure 9 depicts the retrieved images for beach and grounds images, where the first images from top left corner are queries.  Figure 10 depicts the top 20 dinosaurs and horses' images from Corel-1K image dataset, where top upper left corner images are the queries. The results may be changes if different query images are selected but in overall categories, dinosaur, and horse images have given the best results. In case of lowest results, the beach and mountain image categories are shown in Figure 11, where blue color cross symbol × represents the nonrelevant images.

### 4.4. Comparative Study and Analysis

To check the relative efficacy of the proposed CBIR method, the authors have compared the results with ten relative state of art CBIR schemes [24–33]. The discussion and analysis with our proposed scheme is as follows. The most of the above discussed papers have very good retrieval results but they have many limitations such as high dimensional feature, retrieval speed descriptors, and accuracy. However, our proposed method has a high retrieval speed and low dimensional feature descriptors with satisfactory results in terms of average precision, recall, and F-score. The proposed method is compared in terms of average precision (@*P*_*avg*_), recall (@*R*_*avg*_), and F-score (@*F*_*avg*_) with the above-discussed methods and it is shown in Tables 5–7, respectively. In the CBIR schemes [24–27, 29] mountain category images have the minimum retrieval accuracy while beach image category in [28, 32] schemes has the minimum accuracy in terms of @*P*_*avg*_, @*R*_*avg*_, and @*F*_*avg*_. The elephant images have the minimum accuracy in CBIR scheme [30] while scheme [31] has the lowest accuracy for monuments image category. Lastly, the foods image category has the lowest accuracy in CBIR scheme [33]. The most of images in mountain, beach, elephant, and monuments categories have blue color contents which are the mixed with each other while the food category images have very complex structures, so it is very difficult to distinguish actual image contents to classify such categories. So, strong feature extraction methods are required. In our proposed method, mountain images have the lowest accuracy i.e., also 72.70% precision which also acceptable. The overall means of @*P*_*avg*_, @*R*_*avg*_, and @*F*_*avg*_ are 86.89%, 17.38%, and 28.96%, respectively, for the proposed scheme. As compare to the other state of art methods, the proposed scheme has generated the good retrieval results in most of the categories. It is evident that our proposed scheme has better retrieval results than the existing CBIR schemes.

## 5. Conclusion

A novel content-based image retrieval strategy focusing on key color and texture feature descriptors is presented in this research. Color information has been retrieved from a quantized image using color moments. Using the Sobel edge detection algorithm and the GLCCM approach, texture information based on DCT is calculated. The single feature descriptor has a relatively small dimension, allowing it to represent an original image in a compact format without sacrificing retrieval performance and increasing the system's retrieval speed. The experimental obtained results are [31] compared to certain state-of-the-art algorithms using two benchmark datasets, and it is concluded that the average recall rate [45], precision rate, and F-score rate are extremely efficient. In the future, various deep learning-based features could be used to execute the presented feature extraction technique, which could be useful in a variety of real-world applications.

## Figures and Tables

**Algorithm 1:**
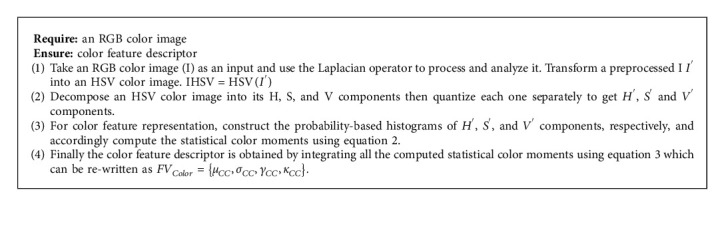
Color information extraction.

**Algorithm 2:**
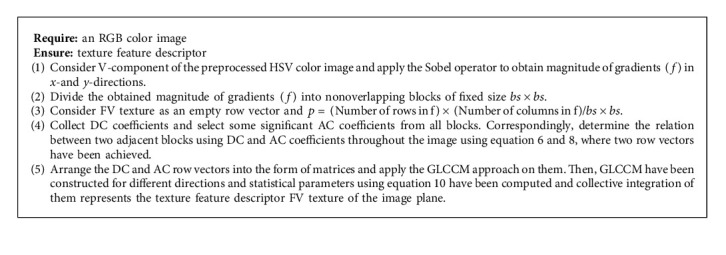
Texture information extraction.

**Table 1:** @*R*_*avg*_, @*P*_*avg*_, and @*F*_*avg*_ for top 10 retrieved images taken from UC Merced land-use dataset.

Image class name	Top 10 images					
	Without preprocessing			With preprocessing		
	@*R*_*avg*_	@*P*_*avg*_	@*F*_*avg*_	@*R*_*avg*_	@*P*_*avg*_	@*F*_*avg*_
Agricultural	8.46	84.60	15.38	9.10	91.00	16.55
Runway	5.66	56.60	10.29	8.65	86.50	15.73
Roads	7.82	78.20	14.22	9.04	90.40	16.44
Grounds	8.50	85.00	15.45	7.93	79.30	14.42
Beach	6.84	68.40	12.44	7.55	75.50	13.73
Residential	7.75	77.50	14.09	8.17	81.70	14.85
Forest	9.02	90.20	16.40	9.23	92.30	16.78
Chaparral	9.05	90.52	16.46	9.95	99.50	18.09
Harbor	8.59	85.9	15.62	9.29	92.90	16.89
Mean	**7.97**	**79.66**	**14.49**	**8.77**	**87.70**	**14.96**

The bold values concludes the mean representation.

**Table 2:** @*R*_*avg*_, @*P*_*avg*_ , and @*F*_*avg*_ for top 20 retrieved images taken from UC Merced land-use dataset.

Image class name	Top 20 images					
	Without preprocessing			With preprocessing		
	@*R*_*avg*_	@*P*_*avg*_	@*F*_*avg*_	@*R*_*avg*_	@*P*_*avg*_	@*F*_*avg*_
Agricultural	14.32	71.60	23.87	16.91	84.55	28.18
Runway	11.59	57.95	19.32	15.46	77.30	25.77
Roads	13.57	67.85	22.62	17.07	85.35	28.45
Grounds	12.94	64.70	21.57	14.28	71.40	23.80
Beach	13.86	69.30	23.10	14.71	73.55	24.52
Residential	15.37	76.85	25.62	16.20	81.00	27.00
Forest	16.95	84.75	28.25	18.05	90.25	30.08
Chaparral	18.03	90.15	30.05	19.90	99.50	33.17
Harbor	16.94	84.70	28.23	17.35	86.75	28.92
Mean	**14.84**	**74.21**	**24.74**	**16.66**	**83.29**	**27.76**

The bold value indicates the mean representation.

**Table 3:** @*R*_*avg*_, @*P*_*avg*_ , and @*F*_*avg*_ for top 10 retrieved images taken from Corel-1K dataset using with and without preprocessed technique.

Image class name	Top 10 images					
	Without preprocessing			With preprocessing		
	@*R*_*avg*_	@*P*_*avg*_	@*F*_*avg*_	@*R*_*avg*_	@*P*_*avg*_	@*F*_*avg*_
Beaches	6.45	64.50	11.73	7.91	79.10	14.38
Buses	9.04	90.40	16.44	9.94	99.40	18.07
Dinosaurs	9.86	98.60	17.93	9.98	99.80	18.15
Elephants	7.83	78.30	14.24	8.75	87.50	15.91
Flowers	8.79	87.90	15.98	9.62	96.20	17.49
Foods	8.93	89.30	16.24	9.48	94.80	17.24
Horses	8.86	88.60	16.11	9.96	99.60	18.11
Monuments	7.75	77.50	14.09	8.75	87.50	15.91
Mountains	6.05	60.50	11.00	7.85	78.50	14.27
People	8.21	79.70	14.92	9.87	98.70	17.95
Mean	**8.18**	**81.53**	**14.87**	**9.21**	**92.11**	**16.75**

The bold value indicates the mean representation.

**Table 4:** @*R*_*avg*_, @*P*_*avg*_ , and @*F*_*avg*_ for top 20 retrieved images taken from Corel-1K dataset using with and without preprocessed technique.

Image class name	Top 20 images					
	Without preprocessing			With preprocessing		
	@*R*_*avg*_	@*P*_*avg*_	@*F*_*avg*_	@*R*_*avg*_	@*P*_*avg*_	@*F*_*avg*_
Beaches	12.18	63.30	20.43	15.41	77.05	25.68
Buses	15.80	85.32	26.66	18.71	93.55	31.18
Dinosaurs	18.01	90.05	30.02	19.05	95.25	31.75
Elephants	12.01	69.66	20.49	16.83	84.15	28.05
Flowers	17.40	90.48	29.19	18.98	94.90	31.63
Foods	14.59	77.33	24.55	17.04	85.20	28.40
Horses	16.41	85.33	27.53	18.07	90.35	30.12
Monuments	11.60	58.00	19.33	16.75	83.75	27.92
Mountains	10.82	59.51	18.31	14.54	72.70	24.23
People	15.38	81.51	25.88	18.39	91.95	30.65
Mean	**14.42**	**76.05**	**24.24**	**17.38**	**86.89**	**28.97**

The bold value indicates the mean representation.

**Table 5:** Comparative results in terms of average precision for top 20 retrieved images for Corel-1K dataset with some state of art CBIR schemes.

Image category	Shrivastava and Tyagi [24]	Dubey et al. [25]	Mistry et al. [26]	Irtaza et al. [27]	Ahmed et al. [28]	Garg and Dhiman [30]	Vimina and Divya [29]	Varish and Singh [31]	Kumar et al. [32]	Joseph et al. [33]	Proposed scheme
People	74.80	75.00	62.00	83.00	90.00	81.20	72.50	60.00	80.00	81.00	91.95
Beaches	58.20	55.00	71.00	72.00	60.00	96.56	52.75	55.00	65.00	78.00	77.05
Monuments	62.10	67.00	53.00	86.00	90.00	78.20	52.70	50.00	65.00	80.00	83.75
Buses	80.20	95.00	77.00	100.00	75.00	83.30	93.95	95.00	100.00	81.00	93.55
Dinosaurs	100.00	97.00	89.00	97.00	100.00	82.20	99.45	100.00	100.00	100.00	95.25
Elephants	75.10	63.00	55.00	82.00	70.00	77.50	52.40	45.00	70.00	81.00	84.15
Flowers	92.30	93.00	89.00	86.00	90.00	86.30	86.65	70.00	95.00	89.00	94.90
Horses	89.60	89.00	55.00	82.00	100.00	86.10	83.75	95.00	100.00	95.00	90.35
Mountains	56.10	45.00	52.00	69.00	70.00	86.30	43.15	80.00	70.00	68.00	72.70
Foods	80.30	70.00	55.00	90.00	90.00	90.20	71.05	65.00	90.00	60.00	85.20
Mean	**76.90**	**74.90**	**65.80**	**84.70**	**83.50**	**84.79**	**71.04**	**71.50**	**83.50**	**81.30**	**86.89**

The bold value indicates the mean representation.

**Table 6:** Comparative results in terms of average recall for top 20 retrieved images for Corel-1K dataset with some state of art CBIR schemes.

Image category	Shrivastava and Tyagi [24]	Dubey et al. [25]	Mistry et al. [26]	Irtaza et al. [27]	Ahmed et al. [28]	Garg and Dhiman [30]	Vimina and Divya [29]	Varish and Singh [31]	Kumar et al. [32]	Joseph et al. [33]	Proposed scheme
People	14.96	15.00	12.40	16.60	18.00	16.24	14.50	12.00	16.00	16.20	18.39
Beaches	11.64	11.00	14.20	14.40	12.00	19.31	10.55	11.00	13.00	15.60	15.41
Monuments	12.42	13.40	10.60	17.20	18.00	15.64	10.54	10.00	13.00	16.00	16.75
Buses	16.04	19.00	15.40	20.00	15.00	16.66	18.79	19.00	20.00	16.20	18.71
Dinosaurs	20.00	19.40	17.80	19.40	20.00	16.44	19.89	20.00	20.00	20.00	19.05
Elephants	15.02	12.60	11.00	16.40	14.00	15.50	10.48	9.00	14.00	16.20	16.83
Flowers	18.46	18.6	17.80	17.20	18.00	17.26	17.33	14.00	19.00	17.80	18.98
Horses	17.92	17.80	11.00	16.40	20.00	17.22	16.75	19.00	20.00	19.00	18.07
Mountains	11.22	9.00	10.40	13.80	14.00	17.26	8.63	16.00	14.00	13.60	14.54
Foods	16.06	14.00	11.00	18.00	18.00	18.04	14.21	13.00	18.00	12.00	17.04
Mean	**15.38**	**14.98**	**13.16**	**16.94**	**16.70**	**16.96**	**14.21**	**14.30**	**16.70**	**16.26**	**17.38**

**Table 7:** Comparative results in terms of average F-score for top 20 retrieved images for Corel-1K dataset with some state of art CBIR schemes.

Image category	Shrivastava and Tyagi [24]	Dubey et al. [25]	Mistry et al. [26]	Irtaza et al. [27]	Ahmed et al. [28]	Garg and Dhiman [30]	Vimina and Divya [29]	Varish and Singh [31]	Kumar et al. [32]	Joseph et al. [33]	Proposed scheme
People	24.93	25.00	20.67	27.67	30.00	27.07	24.17	20.00	26.67	27.00	30.65
Beaches	19.40	18.33	23.67	24.00	20.00	32.19	17.58	18.33	21.67	26.00	25.68
Monuments	20.70	22.33	17.67	28.67	30.00	26.07	17.57	16.67	21.67	26.67	27.92
Buses	26.73	31.67	25.67	33.33	25.00	27.77	31.32	31.6	33.33	27.00	31.18
Dinosaurs	33.33	32.33	29.67	32.33	33.33	27.40	33.15	33.33	33.33	33.33	31.75
Elephants	25.03	21.00	18.33	27.33	23.33	25.83	17.47	15.00	23.33	27.00	28.05
Flowers	30.77	31.00	29.67	28.67	30.00	28.77	28.88	23.33	31.67	29.67	31.63
Horses	29.87	29.67	18.33	27.33	33.33	28.70	27.92	31.67	33.33	31.67	30.12
Mountains	18.70	15.00	17.33	23.00	23.33	28.77	14.38	26.67	23.33	22.67	24.23
Foods	26.77	23.33	18.33	30.00	30.00	30.07	23.68	21.67	30.00	20.00	28.40
Mean	**25.63**	**24.97**	**21.93**	**28.23**	**27.83**	**28.26**	**23.68**	**23.83**	**27.83**	**27.10**	**28.96**

The bold value indicates the mean representation.

## Data Availability

No data were used to support this study.
